# A Fatal Case of Multidrug-Resistant Pleural Nocardiosis by Nocardia otitidiscaviarum in an Immunosuppressed Patient: A Case Report and Literature Review

**DOI:** 10.7759/cureus.52071

**Published:** 2024-01-10

**Authors:** Rahul Ranjan, Raunak Bir, Jayanthi Gunasekaran, Vishwanath S Yadav, Rajiv M Gupta

**Affiliations:** 1 Department of Microbiology, Employees' State Insurance Corporation (ESIC) Medical College and Hospital, Faridabad, IND

**Keywords:** multidrug-resistant bacteria, steroids, branching filamentous rods, amikacin, meropenem, cotrimoxazole, human immunodeficiency virus, immunosuppressed patient, nocardia otitidiscaviarum, pleural nocardiosis

## Abstract

Nocardiosis is known as an opportunistic infection in immunocompromised hosts. We present to you a case of pleural nocardiosis in a 38-year-old male patient who was a chronic smoker and presented with a left-sided pleural effusion. He was a known case of thrombocytopenia due to immune thrombocytopenia (ITP) and was on steroid therapy. On admission, he was found to be positive for HIV. Pleural fluid was sent to microbiology, where acid-fast staining with 1% sulfuric acid (H_2_SO_4_)showed acid-fast branching filamentous rods and cultures grew *Nocardia,* which was resistant to ampicillin, ceftriaxone, imipenem, cotrimoxazole, erythromycin, tetracycline, and susceptible to amikacin, linezolid, and levofloxacin. The isolate was identified as *Nocardia otitidiscaviarum* using 16S rRNA gene sequencing. Culture from the chest wall drain grew* Escherichia coli* and *Stenotrophomonas maltophilia.* Subsequently, the patient developed sepsis, and paired blood cultures grew *Candida guilliermondii*. Unfortunately, the patient could not survive despite aggressive efforts and died after 40 days of admission.

## Introduction

*Nocardia spp.* are gram-positive bacteria found in soil, water, and decaying vegetation [1]. Human immunodeficiency virus (HIV) infection, malignancy, diabetes mellitus, transplantation, and corticosteroid use predispose patients to nocardiosis, although infection can occur in immunocompetent hosts. [2] The clinical manifestations of nocardiosis can be diverse, but the most common sites are the lungs, where the infection occurs by inhalation or direct inoculation of *Norcadia*. [3] *Nocardia otitidiscaviarum*, first named *Nocardia caviae,* was first reported in humans in the 1960s [4]. *Nocardia otitidiscaviarum* was previously reported in 1924 when it was isolated from a guinea pig's middle ear. [4]

## Case presentation

A 38-year-old male patient came to the emergency department with complaints of chest pain on the left side, shoulder pain, and dysphagia for two days. He denied shortness of breath, fever, and a productive cough. The patient had lost 10 kg in the last three months. The patient was a farmer by profession. The patient was a chronic smoker of tobacco (40 pack years) and an alcoholic for the past 10 years and consumed about 150-250 ml/day of alcohol once a day. There was no history of tuberculosis, diabetes mellitus, or hypertension. He had a known history of thrombocytopenia due to hemophagocytic lymphohistiocytosis (HLH)/ immune thrombocytopenia (ITP) and was on steroid therapy.

**Figure 1:**
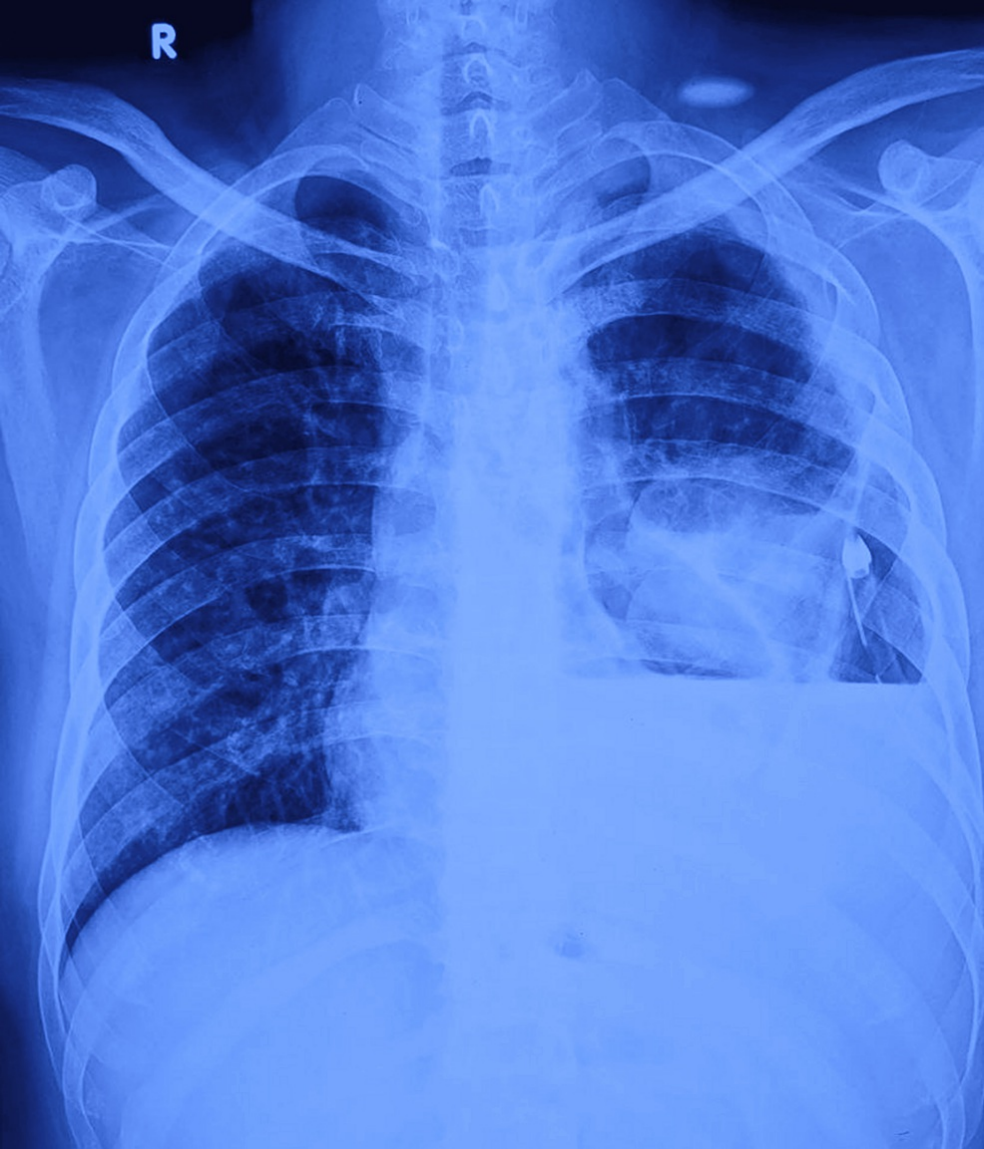
The chest X-ray showed left homogeneous opacity.

On examination, he was conscious and oriented to time, place, and person. He was afebrile, his blood pressure was 117/74 mmHg, his pulse was 97 beats per minute, he was hemodynamically stable, and his oxygen saturation was 97% on room air. On chest auscultation, left lung air entry was decreased. The chest X-ray showed left homogeneous opacity and consolidation suggestive of parapneumonic pleural effusion or empyema (Figure 1).

High-resolution computed tomography (HRCT) of the chest was suggestive of a left cavitary and consolidatory lesion with effusion. Based on these findings, a left-side flexible chest tube was inserted for the drainage of pleural effusion, and the tube was left there as the effusion was not resolving.

Based on the clinico-radiological findings and biochemical analysis of the pleural fluid, which showed lactate dehydrogenase (LDH) of 820 IU/L, sugar of 23 g/dl, and total protein of 33 g/dl, tuberculosis was suspected, and anti-tubercular treatment was started. Intravenous (IV) piperacillin/tazobactam 4.5 g four times a day (QID), IV clindamycin 600 mg three times a day (TDS), and oral fluconazole 150 mg once a day (OD) were administered empirically after the samples were collected for culture.

**Figure 2:**
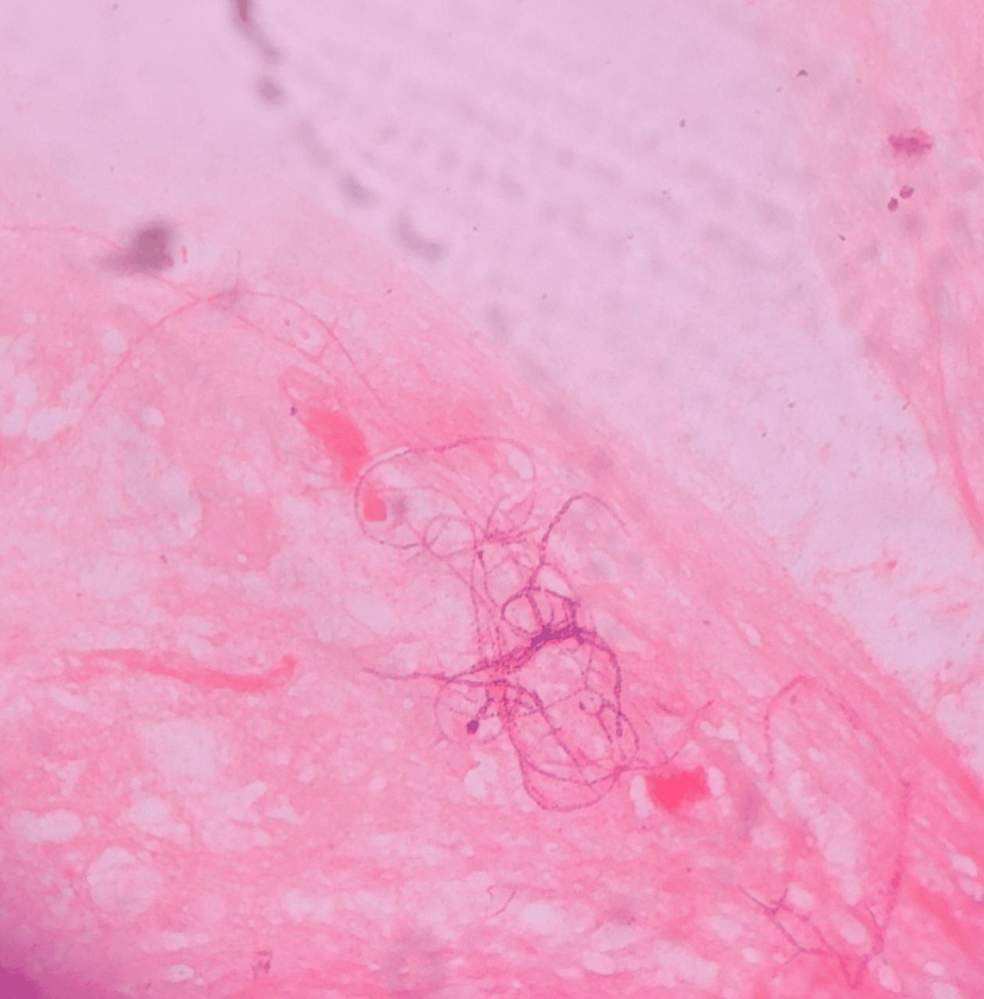
Gram stain of the pleural fluid showed gram-positive beaded branching filamentous bacilli.

The patient was diagnosed as HIV-reactive by third-generation HIV testing. The patient's CD4 count was 42 cells/ul (CD 4% = 5). Pleural fluid was sent for microbiological examination. On the gram stain, branching filamentous beaded gram-positive bacilli were seen (Figure 2).

**Figure 3:**
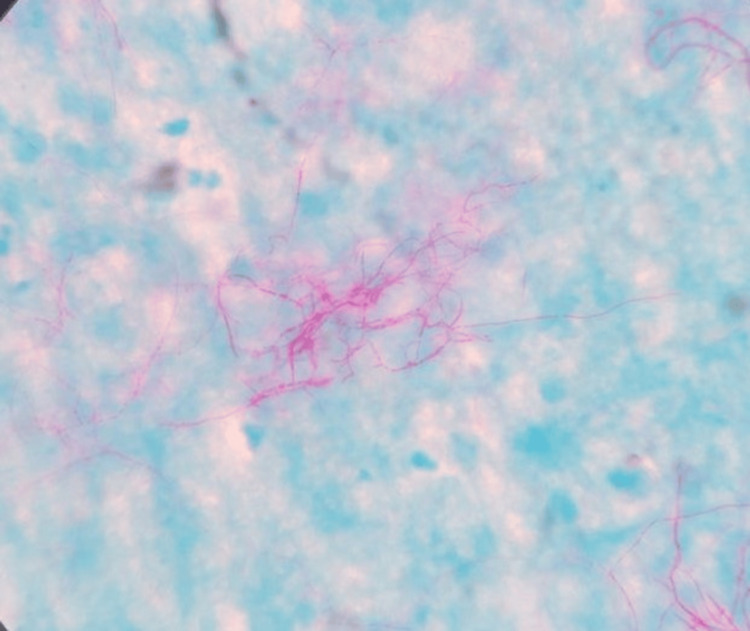
Modified Ziehl-Neelsen stain (1% sulphuric acid (H2SO4)) of the pleural fluid, showing pink-colored branching acid-fast filamentous bacilli suggestive of Nocardia species.

Modified Ziehl-Neelsen stain (1% sulfuric acid (H_2_SO_4_)) from the sample demonstrated pink-colored branching acid-fast filamentous bacilli suggestive of *Nocardia *species (Figure 3).

No pathogenic microorganism could be isolated from sputum after 48 hours of incubation. Blood cultures (one set) were negative after five days of incubation. A 10% potassium hydroxide mount of pleural fluid and sputum was done, and no fungal elements were seen. *Mycobacterium tuberculosis* complex was not detected by the cartridge-based nucleic acid amplification test (CBNAAT) of the sputum and pleural fluid. On stool examination by saline mount, iodine mount, and modified acid-fast stain with 1% H_2_SO_4_, no ova, cyst, or oocyst of parasites were found.

**Figure 4:**
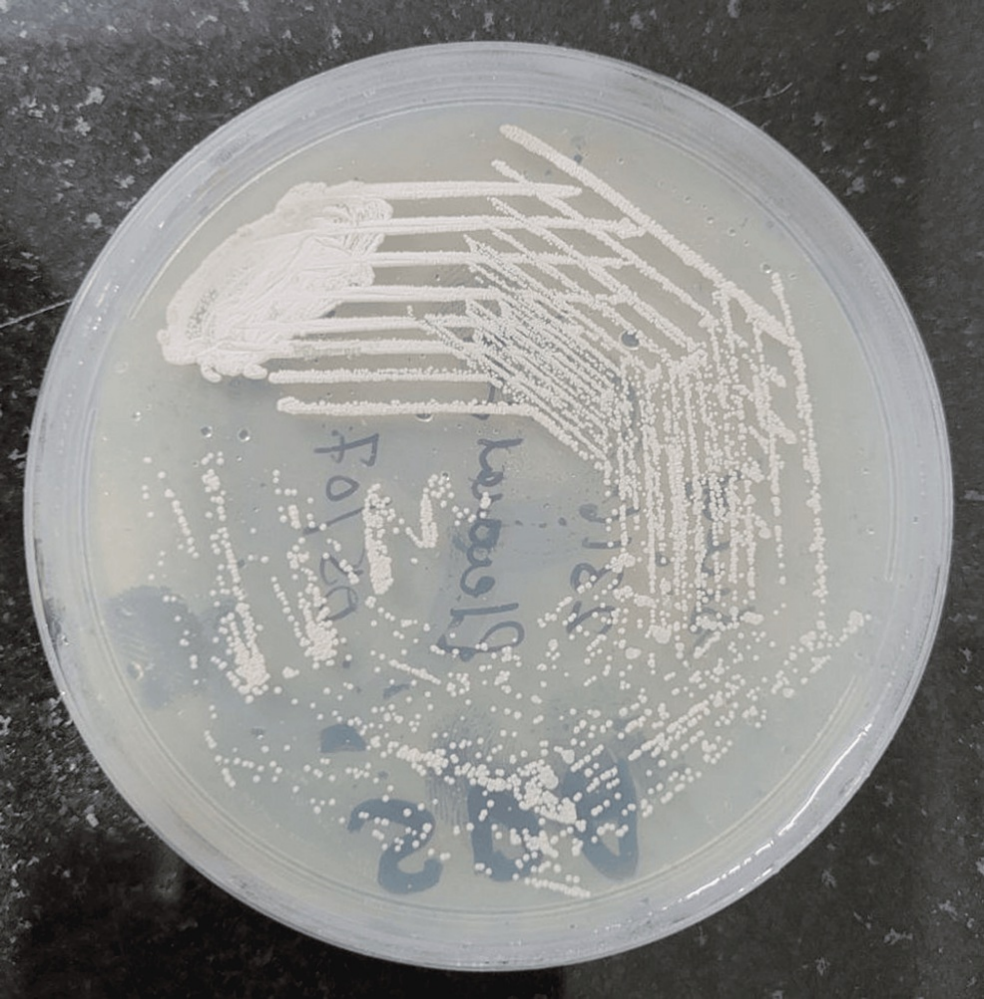
Chalky white dry colonies on Sabouraud dextrose agar

**Figure 5:**
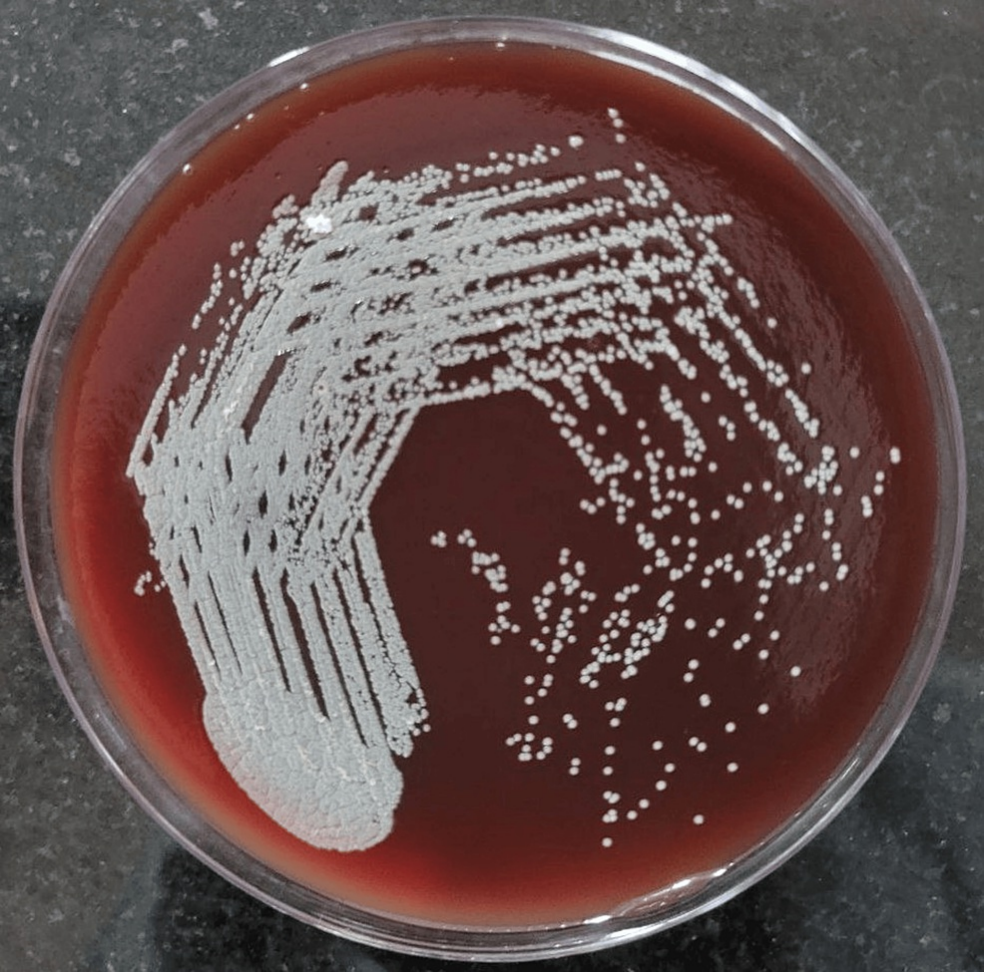
Chalky white dry colonies on blood agar

A culture of pleural fluid yielded chalky white dry colonies on Sabouraud dextrose agar (Figure 4) and blood agar (Figure 5) morphology suggestive of *Nocardia spp.*, and the colonies also showed pitting.

The patient was started empirically on oral trimethoprim-sulfamethoxazole (TMP-SMX) double strength in view of nocardiosis while awaiting an antimicrobial susceptibility testing report. The isolate was identified as *Nocardia otitidiscaviarum* by matrix-assisted laser desorption ionization-time of flight (MALDI-TOF) mass spectrometry with a 99.9% confidence value and 16S rRNA sequencing (GenBank accession number: OR533575).

Antimicrobial susceptibility testing using Kirby-Bauer disc diffusion testing was done on blood agar, and the following drugs were tested: amikacin (30 ug), linezolid (30 ug), levofloxacin (5 ug), ampicillin (10 ug), ceftriaxone (30 ug), imipenem (10 ug), cotrimoxazole (25 ug), erythromycin (15 ug), and tetracycline (30 ug). The isolate was resistant to ampicillin, ceftriaxone, imipenem, cotrimoxazole, erythromycin, and tetracycline. The isolate was susceptible to amikacin, linezolid, and levofloxacin. The patient was started on IV levofloxacin 750mg OD and IV amikacin 750mg OD. Anti-retroviral treatment was initiated.

Other significant laboratory findings were that the patient's CD4 count was 42 cells/ul (CD 4% = 5), and subsequently he developed anemia (hemoglobin: 4.9 g/dL), leukopenia (WBC count: 580/uL), hyperbilirubinemia (total bilirubin: 1.47 mg/dl, direct bilirubin: 1.07 mg/dl, indirect bilirubin: 0.40 mg/dl, serum glutamic-oxaloacetic transaminase (SGOT): 50 U/L, serum glutamic pyruvic transaminase (SGPT): 41 U/L, alkaline phosphatase: 163 IU/L, and creatinine: 1.2 mg/dl), and hepatic encephalopathy, and he was shifted to the ICU.

To cover the potential hospital-acquired pathogens, IV meropenem 1 gm TDS and IV teicoplanin 400 mg twice a day (BD) were added empirically. Repeat pleural fluid culture after five days grew *Nocardia otitidiscaviarum*, carbapenem-resistant *Acinetobacter baumannii *complex susceptible to minocycline and colistin, and carbapenem-resistant *Escherichia coli* susceptible to amikacin, gentamicin, tigecycline, and colistin.

Culture from a chest wall drain tube grew carbapenem-resistant *Escherichia coli* susceptible to amikacin, gentamicin, tigecycline, and colistin, and *Stenotrophomonas maltophilia* susceptible to levofloxacin, minocycline, and TMP-SMX. Subsequently, the patient developed sepsis. Paired blood culture samples grew *Candida guilliermondii*, which was susceptible to voriconazole, caspofungin, and micafungin.

After 40 days of admission, even after continuous efforts and treatment, unfortunately, the patient could not survive.

## Discussion

Our case report presents a rare instance of empyema caused by *Nocardia otitidiscaviarum*. As it is described as an opportunistic pathogen in individuals with weakened immune systems. Patients with various immunocompromised states are at higher risk of acquiring infections from *Nocardia otitidiscaviarum.* [4] Similarly, in our case, the patient was HIV-infected and was on steroids for ITP.

Sulphonamides were used for the treatment of *Nocardia *infections in the past, and treatment was effective in most of the patients. However, when we handle a patient infected with *Nocardia otitidiscaviarum*, it is very important to do the antimicrobial susceptibility testing before starting the treatment, as previous studies have reported resistance to beta-lactam drugs like ampicillin, amoxicillin-clavulanic acid, and imipenem, along with variable susceptibility to sulphonamides.

This case report highlights the importance of early diagnosis, species identification, and susceptibility testing for choosing the right treatment for the patient in order to improve the outcome. The treatment of *Nocardia *infections depends on the susceptibility pattern, and long-term treatment is also required depending on the severity of the underlying systemic disease. Careful consideration needs to be given when a case of nocardiosis is being treated.

We have summarized 10 recent case reports of *Nocardia otitidiscaviarum* reported in the literature in Table 1 below.

**Table 1:** Summary of 10 recent case reports of Nocardia otitidiscaviarum reported in the literature.

Study, year, and place	Age/sex-associated risk factors/underlying disease	Site of infection and clinical presentation	Antimicrobial susceptibility: susceptible and resistant	Treatment given	Outcome
Present case, 2023, Faridabad, India	38-year-old male; HIV, thrombocytopenia, and on steroid therapy	In the pleural cavity. Left chest pain, left shoulder pain, and dysphagia	Sensitive: amikacin, linezolid, and levofloxacin; Resistant: ampicillin, ceftriaxone, imipenem, trimethoprim-sulfamethoxazole (TMP-SMX), erythromycin, and tetracycline	Levofloxacin and amikacin	Died
Barry M et al., 2022, Riyadh, Saudi Arabia [4]	A 37-year-old female with metastatic breast cancer	Intra-cranial presentation. Skin seizures and multiple skin nodules	Sensitive: amikacin, linezolid, moxifloxacin, and doxycycline; Resistant: TMP–SMX and Imipenem	Amikacin, linezolid, moxifloxacin, and doxycycline	Died
Parengal J et al., 2021, Doha, Qatar [5]	A 29-year-old female with systemic lupus erythematosus (SLE). Multi-organ involvement, including autoimmune hemolytic anemia, cerebritis, lupus nephritis, cardiomyopathy with an ejection fraction of 33%, and non-specific interstitial pneumonia. On steroid therapy.	Pulmonary organs and skin. Dry cough followed by fever, progressive shortness of breath, generalized body aches, nausea, vomiting, and skin rash.	Sensitive: TMP-SMX, amikacin, ciprofloxacin, moxifloxacin, and linezolid; Resistant: amoxicillin-clavulanate, ceftriaxone, and clarithromycin	Meropenem, TMP-SMX, and amikacin	Recovered
Sah R et al., 2020, Kathmandu, Nepal [6]	61-year-old-male on steroid (1 mg/kg/d) therapy for eight weeks for a recent diagnosis of nephrotic syndrome.	Pulmonary and lymphocutaneous (subcutaneous) infection. Fever along with coughing and swelling of the right thigh.	Sensitive: TMP-SMX, imipenem, amikacin, and linezolid; Resistant: ceftriaxone	Meropenem, amikacin, and TMP-SMX	Recovered
Saksena R et al., 2020, New Delhi, India [7]	Case 1: A 70-year-old female with no predisposing condition.	Pulmonary cough with expectoration, on and off fever, difficulty in breathing, and chest pain.	Sensitive: linezolid, ciprofloxacin, amikacin, and gentamicin; Resistant: TMP-SMX, ampicillin, amoxicillin-clavulanic acid, imipenem, and erythromycin	Injection amoxicillin-clavulanate, tab azithromycin, and tab TMP-SMX	Died
	Case two: A 70-year-old male diagnosed with pulmonary tuberculosis over 10 years ago. Smoking and alcohol consumption off and on	Pulmonary productive cough, fever, and shortness of breath	Sensitive: ciprofloxacin, amikacin, linezolid, and gentamicin; Resistant: erythromycin, ampicillin, amoxicillin-clavulanic acid, imipenem and TMP-SMX.	Inj meropenem, IV colistin, and tab TMP-SMX	Died
Princess et al., 2018, Tamil Nadu, India [8]	A 51-year-old male with hypertension and childhood-onset bronchial asthma requiring chronic steroid therapy	Pulmonary high-grade, intermittent fever and cough with expectoration and breathlessness	Sensitive: amikacin, ciprofloxacin, linezolid, imipenem, and ceftriaxone; Resistant: TMP-SMX and amoxicillin-clavulanate	TMP-SMX + imipenem	Died
Tajima K et al., 2018, Yamagata, Japan [9]	A 66-year-old male with epilepsy and malignant lymphoma	Pulmonary presentation with meninges+fever and vomiting	Sensitive: amikacin, gentamicin, linezolid, and TMP-SMX; Resistant: ampicillin, ceftriaxone, and ciprofloxacin,	Linezolid and TMP-SMX	Recovered
Thirouvengadame S et al., 2017, Puducherry, India [10]	A 66-year-old male with no predisposing condition	Pulmonary presentation with intermittent cough and breathlessness, and loss of weight and appetite	Sensitive : amikacin, gentamicin, TMP-SMX, ceftriaxone, imipenem, and linezolid; Resistant: amoxicillin-clavulanic acid	Oral TMP-SMX	Recovered
Liu C et al., 2017, Sichuan, China [11]	A 58-year-old male cotton farmer; hepatitis B virus carrier and a smoker	Pulmonary presentation with recurrent fever, productive cough, and dyspnea	Not performed	TMP-SMX + amikacin + imipenem	Died
Sadamatsu H et al., 2017, Saga, Japan [12]	A 72-year-old female with influenza A and bronchial asthma. She was on inhaled corticosteroids.	Pulmonary presentation with fever, malaise, and productive cough	Sensitive: TMP-SMX, minocycline, levofloxacin, and imipenem; Resistant: ceftriaxone	Minocycline	Recovered
Deepa R et al., 2016, Chennai, India [13]	A 14-year-old female with rheumatic heart disease	Pulmonary presentation with difficulty in breathing, cough with expectoration, right-sided chest pain, and low-grade intermittent fever	Sensitive: gentamicin, ciprofloxacin, amikacin, TMP-SMX, tetracycline, imipenem; Resistant: amoxicillin-clavulanate, cefotaxime, and ceftriaxone	Ceftriaxone	Died

## Conclusions

Cases of *Nocardia otitidiscaviarum* ​​​​​​​presented in the past literature show a case fatality rate of more than 50%. The infection can be fatal if it happens to an immunocompromised patient. Therefore, early diagnosis and treatment according to the susceptibility patterns are very important in treating and saving the patient.

## References

[1] Conville PS, Witebsky FG (2015). Nocardia, Rhodococcus, Gordonia, Actinomadura, Streptomyces, and other aerobic actinomycetes. Manual of Clinical Microbiology, 11th Edition.

[2] Chow FC, Marson A, Liu C (2013). Successful medical management of a Nocardia farcinica multiloculated pontine abscess. BMJ Case Rep.

[3] Paige EK, Spelman D (2019). Nocardiosis: 7-year experience at an Australian tertiary hospital. Intern Med J.

[4] Barry M, AlShehri S, Alguhani A (2022). A fatal case of disseminated nocardiosis due to Nocardia otitidiscaviarum resistant to trimethoprim-sulfamethoxazole: case report and literature review. Ann Clin Microbiol Antimicrob.

[5] Parengal J, Alebbi SM, Hamed MM, Alqatami HM, Ben Abid F (2021). Disseminated life threatening Nocardia otitidiscaviarum infection in a young female with newly diagnosed systemic lupus erythematosus, case report and review of literature. IDCases.

[6] Sah R, Khadka S, Neupane S (2020). Disseminated infection with Nocardia otitidiscaviarum in a patient under steroid therapy. Clin Case Rep.

[7] Saksena R, Rynga D, Rajan S (2020). Fatal pulmonary infection by trimethoprim-sulfamethoxazole resistant Nocardia otitidiscaviarum: report of two cases and review. J Infect Dev Ctries.

[8] Princess I, Ebenezer R, Ramakrishnan N, Nandini S (2018). Pulmonary nocardiosis and scrub typhus in an immunocompromised host. J Glob Infect Dis.

[9] Tajima K, Terada T, Okuyama S (2018). Nocardia otitidiscaviarum meningitis in a diffuse large B-cell lymphoma patient with CD4-positive lymphocytopenia and persistent oligoclonal CD8-positive lymphocytes in the peripheral blood. Int J Clin Exp Pathol.

[10] Thirouvengadame S, Muthusamy S, Balaji VK, Easow JM (2017). Unfolding of a clinically suspected case of pulmonary tuberculosis. J Clin Diagn Res.

[11] Liu C, Feng M, Zhu J, Tao Y, Kang M, Chen L (2017). Severe pneumonia due to Nocardia otitidiscaviarum identified by mass spectroscopy in a cotton farmer: A case report and literature review. Medicine (Baltimore).

[12] Sadamatsu H, Takahashi K, Tashiro H, Komiya K, Nakamura T, Sueoka‐Aragane N (2017). Successful treatment of pulmonary nocardiosis with fluoroquinolone in bronchial asthma and bronchiectasis. Respirol Case Rep.

[13] Deepa R, Banu ST, Jayalakshmi G, Parveen JD (2016). Pleuropulmonary nocardiosis due to Nocardia otitidiscaviarum in a debilitated host. Indian J Pathol Microbiol.

